# Metabotyping human endometrioid endometrial adenocarcinoma reveals an implication of endocannabinoid metabolism

**DOI:** 10.18632/oncotarget.10564

**Published:** 2016-07-13

**Authors:** Mariona Jové, Sònia Gatius, Andree Yeramian, Manuel Portero-Otin, Núria Eritja, Maria Santacana, Eva Colas, Maria Ruiz, Reinald Pamplona, Xavier Matias-Guiu

**Affiliations:** ^1^ Department of Experimental Medicine, Universitat de Lleida, IRBLleida, E-25198, Lleida, Spain; ^2^ Department of Pathology and Molecular Genetics/Oncologic Pathology Group, Hospital Universitari Arnau de Vilanova, Universitat de Lleida, IRBLleida, E-25198, Lleida, Spain

**Keywords:** endocannabinoid system, metabolomic profile, endometrioid endometrial carcinoma, mass spectrometry, personalized medicine

## Abstract

Metabolomics, an essential technique in precision medicine, contributes to the molecular fingerprinting of tumours, further helping to understand their pathogenesis. In this work, using a LC-ESI-QTOF-MS/MS platform, we demonstrated the existence of a specific metabolomic signature which could define endometrioid endometrial carcinoma (EEC), arising the endocannabinoid system as a potential pathway involved in EC pathogenesis. Metabolomics could also shed light in the processes involved in myometrial invasion, proposing new targets for possible therapeutic intervention. Consequently, we also described a different metabolomic profile in surface endometrioid carcinoma and myometrial invasive front. We validated pathways disclosed by metabolomics by immunohistochemistry. Specifically, endocannabinoid and purine metabolism could be involved in tumor myometrial invasion.

## INTRODUCTION

Endometrial cancer (EC) is the most common cancer of the female genital tract [1] and overall the fourth most common cancer in developed regions [2]. There are different histologic types with different morphological features, behavior and molecular background. The most characteristic of them are endometrioid (EEC) and serous (SC) carcinomas. EEC is the most frequent type. It accounts for 80% of EC, and it is usually associated with good prognosis, although 20 % of them recur or metastasize [3]. Prognosis relies on a subset of parameters including histological grade, stage, and presence of lympho-vascular invasion. However, it is still difficult to predict behavior based on the microscopic appearance of the tumors; and there is a subset of low-grade EEC, apparently indolent, that may progress.

Next generation sequencing studies provide a more accurate description of the molecular background of EC. The Cancer Genome Atlas Research Network (TCGA) has recently performed an integrating genomic characterization of EC [4]. Exome sequence analysis revealed four groups of tumors. Group 1, with EEC with mutations in POLE, associated with good prognosis. Group 2, including EEC with microsatellite instability and group 3 tumors including EEC with low copy number alterations, both showing similar progression-free survival rates. Group 4 (Serous-like) showed p53 mutations, worse prognosis, and was composed of most (but not all) SC, but also some EEC (many EEC3, but also some EEC1-2). The TCGA study has shown that a small percentage of tumors that are microscopically EEC, show molecular features of SC, and vice versa. These results emphasize the existence of tumors that microscopically and molecularly are located in the grey zone between EEC and SC, and they stress the need for identifying biomarkers of bad prognosis in EEC.

It has been recently suggested that intra-tumor heterogeneity may be important in tumor progression [5–7]. Under the selective pressure of the environment, some tumor clones may overgrowth in competition with other tumor cell subpopulations, and this is particularly important at the invasive front of the tumor. For that reason, understanding the molecular events that occur at the invasive front may provide important information to understand the mechanisms of EC tumor progression.

Recent technological and bioinformatics advances have contributed to “omic” techniques development [8]. Genetics, transcriptomics, proteomics and metabolomics are essential in biomarker research in cancer. Metabolomics allows monitoring the changes in the whole metabolism. The resulting molecules, metabolites are the end-products of gene expression and their levels mirror genomic, transcriptomic, and proteomic fluctuations [9]. Although many metabolomic approaches have been previously used in cancer biomarker discovery, the metabolomic profile of EC is currently unknown.

Recently, metabolomics has been applied to better understand physiopathological processes involved in cancer progression and propose novel biomarkers [10–12]. Based on these premises, in this study, we aimed to characterize potential metabolomic differences between EEC and normal endometrial tissues, and also between different locations in EEC (invasive front and superficial zone). The results show the identification of potential biomarkers of tumor development and progression, as well as metabolic pathways for EEC personalized medicine.

## RESULTS

### Endometrioid carcinoma shows a characteristic metabolomic pattern with endocannabinoid system as a relevant pathway

**Figure 1:**
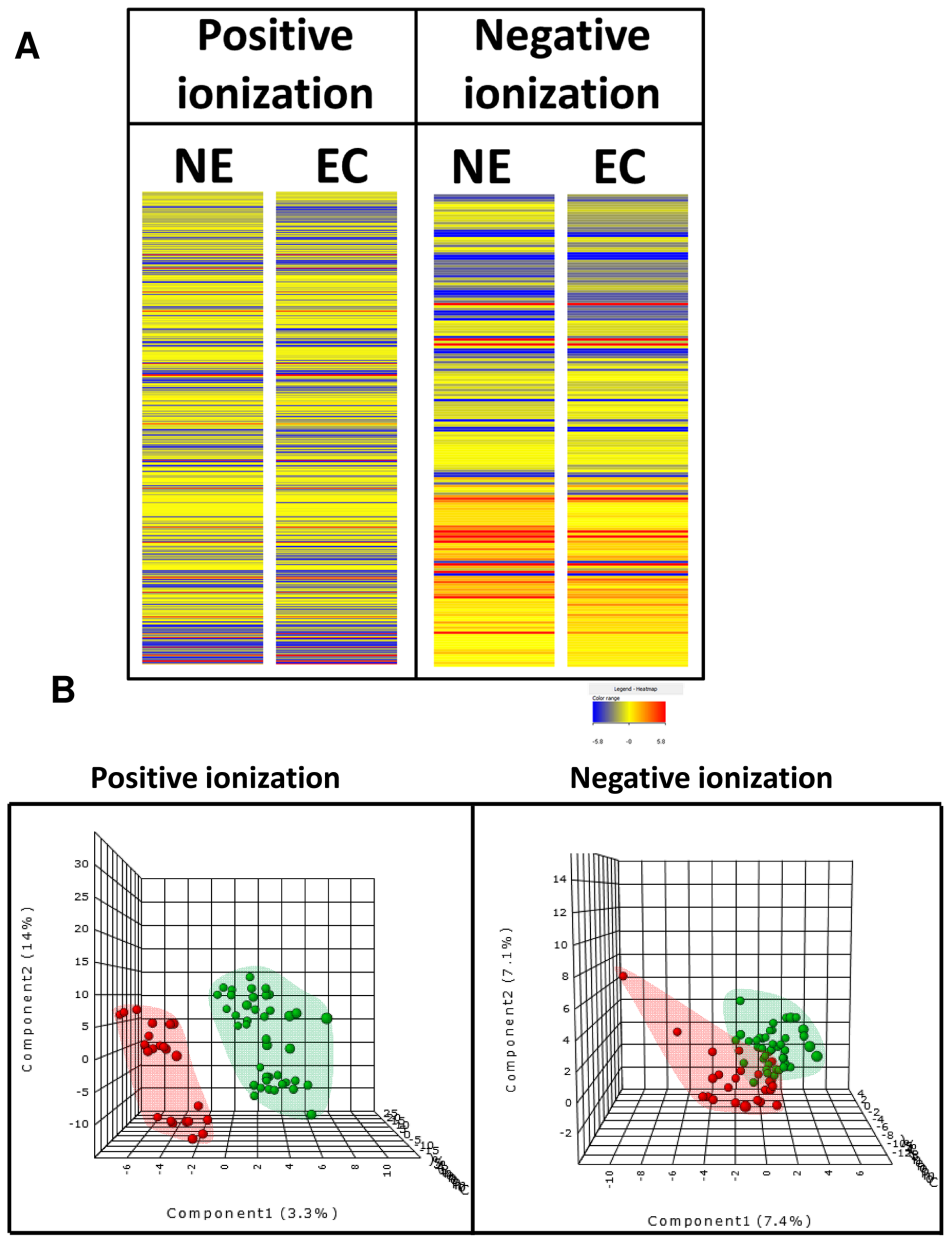
Specific metabolomics profile of normal endometrium (NE) and endometrioid endometrial carcinoma (ECC or EC) **A.** Heat map showing the molecular features (see main text for definition) found in NE and EEC in metabolomic analysis. Individual scale maps for heat intensity are shown below each sample type. **B.** PLS-DA graphs demonstrating different metabolomic profiles. Red spots represents samples of normal endometrium and green spots from endometrioid endometrial carcinoma. PLS-DA model out of bag error is 0.221 for positive and 0.232 for negative ionization.

**Figure 2:**
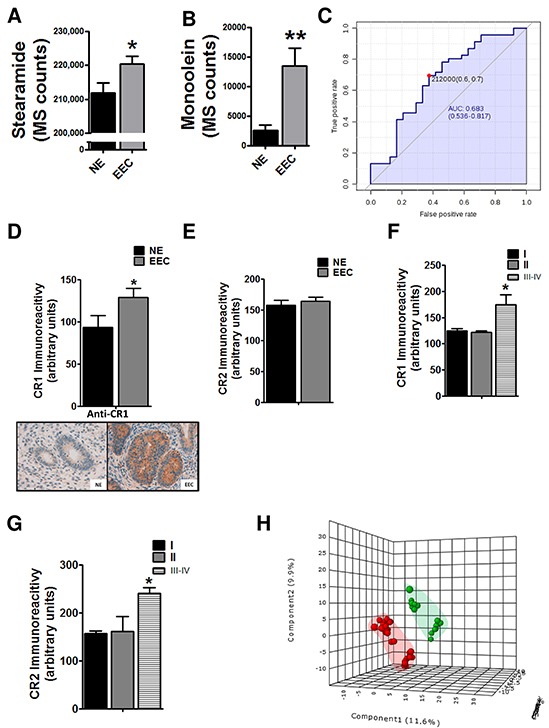
Implication of endocannabinoid pathway in endometrioid endometrial carcinoma (EEC) progression Levels of cannabinoid related metabolites, the stearamide **A.** and the monoolein **B.** are increased in EEC samples. **C.** Predictive power of stearamide in EEC determination. * P<0.05 **D.** The levels of cannabinoid receptor 1 (CR1) are increased in ECC tissue samples whereas there are not differences in CR2 levels **E.** Increased levels of CR1 **F.** and CR2 **G.** in stages III-IV of EEC. **H.** PLS-DA graphs demonstrating different metabolomic profiles in EEC, grades 1-2 and 3. Blue spots represents normal endometrium samples (NE), red spots endometrioid endometrial carcinoma grades 1 and 2 and green spots from endometrioid endometrial carcinoma grade 3. * P<0.05

The first aim of this work was to analyze metabolomic differences between EEC (considering together surface endometrial carcinoma (SEC) and myometrial invasive front (MIF)) and normal endometrium NE samples. To do this, we applied a non-targeted metabolomics approach focusing on the profiles of low molecular weight (*m*/*z* < 3000) ionizable molecules and we detected 717 molecular features, according to the parameters described in the materials and methods section. In order to analyze whole metabolome we selected those molecules which were present at least in 50 % of the samples in any group. Heatmap analyses suggested differences in metabolomic profiles between NE and EEC samples, both in positive and in negative ionization modes (Figure 1A). Then, we applied Partial Least Square Discriminating Analysis (PLS-DA) and the differences were confirmed (Figure 1B) indicating the existence of a specific metabolomic signature for each condition. In order to control overfitting we employed two different techniques: we evaluated permutation tests for validation of PLS-DA and we used an alternative technique for multivariate analyses, random forest analyses. The existence of a metabolomics signature was supported by both analyses, which lead to a significant permutation test (p<0.02) for negative ionized molecules and an overall error of 0.22 (Supplementary Figure S1A) in both cases for random forest analyses. Finally, univariate statistics (Student T Test) was applied and we found 53 statistically different molecules (p<0.05) (Supplementary DataSet1). Among them, we could identify (based on exact mass, retention time, isotopic distribution and/or MS/MS spectrum) stearamide, monoolein, hypoxanthine and 1,2-dihexadecanoyl-sn-glycerol. Two of them act as endocannabinoids (stearamide and monoolein) and their levels are increased in EEC samples (Figure 2A and 2B). Then, in order to define the capacity of these molecules as biomarkers, receiver operating characteristic (ROC) curves were performed using metabolites present in at least 50% of the samples in the same group (NE and EEC) and stearamide arose as one with best scores (Figure 2C).

**Figure 3:**
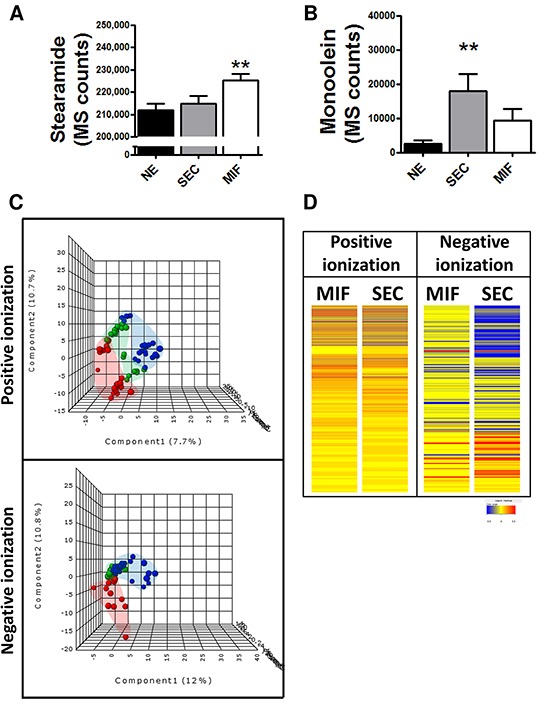
Specific metabolomic profile of tumor cells at the invasive front (MIF) and the cells that are located in the surface of the tumor (SEC) Differential levels of stearamide **A.** and monoolein **B.** according to tumor depth. *P<0.05 **C.** PLS-DA graphs demonstrating different metabolomic profiles. Red spots represents normal endometrium samples, blue spots samples from surface endometrioid carcinoma and green spots from myometrial invasive front.PLS-DA model out of bag error is 0.261 for positive and 0.377 for negative ionization **D.** Heat map showing the molecular features (see main text for definition) found myometrial invasive front (MIF) and surface endometrioid carcinoma (SEC) in metabolomic analysis. Individual scale maps for heat intensity are shown below each sample type.

Endocannabinoid system regulates cell proliferation, differentiation and cell survival of different cell types, and it has been described that modulates migration of endometrial cells [13, 14]. So, to further validate the potential implication of endocannabionoid system in EEC we analyzed, using immunohistochemistry, the levels of CR1 and CR2. The results showed that the CR1 levels were increased in EEC comparing to NE (Figure 2D), in contrast to CR2 expression (Figure 2E). Interestingly, when immunoexpression was analyzed taking into account EEC stages both CR1 and CR2 were increased in samples from stages III-IV, suggesting a relationship between endocannabinoids and aggressive behavior (Figure 2F and 3G). Another parameter highly associated with aggressive behavior is the EEC histological grade, which is based on the amount of solid architectural pattern. In this line, whole metabolomics differences were studied discovering different signatures for low grade (EEC grade 1 and 2) and high grade (EEC grade 3), although not good permutation test was obtained (p≥0.05) (Figure 2H). Further, the out of bag error obtained from random forest analyses classification was 0.36 for positive and 0.43 for negative ionization modes (Supplementary Figure S1C). Among statistically different molecules found between low and high grade we could identified (basing on exact mass, retention time and isotopic distribution) the taurine and erythritol (decreased in grade III) and oleamide (increased in grade III) and (DataSet1). Interestingly, oleamide is an amide of the fatty acid oleic acid which has the ability to bind to the endocannabinoid receptor CR1.

### Surface endometrioid carcinoma and myometrial invasive front differ in metabolomic profile

Due to the potential differences in cell metabolism related to tumor invasion, we analyzed whether changes observed in endocannabinoid metabolism were influenced by this. So, the levels of the two endocannabinoids disclosed in the previous approach as well as CR1 expression were analyzed. We found higher levels of stearamide in MIF but higher levels of monoolein in SEC tissue (Figure 3A and 3B). No differences in CR1 immunoexpression were found when we compared superficial tumor tissue and myoinvasive front (p=0.5; Fold Change=1.04). Due to sharp differences observed between SEC and MIF we analyzed whole metabolome and a characteristic metabolite profiling was observed (Figure 3C and 3D).

**Figure 4:**
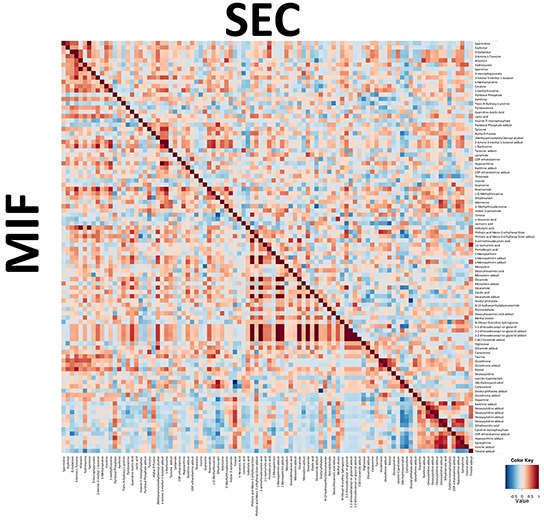
Integration of metabolic correlations of of tumor cells at the invasive front (MIF) and the cells that are located in the surface of the tumor (SEC) The pixel maps were derived from correlation analyses between different identified metabolites. The cutoff value of 0.6 was applied to the absolute PC value for displaying the correlations between metabolites, shown as a color-coded pixel map (gradient of red colors for positive values and gradient of blue colors for negative values).

The same approximation explained before was used to identify metabolomic differences between SEC and MIF samples. Using a non-targeted approach and focusing on the metabolite profiles of low molecular weight (*m*/*z* < 3000) ionizable molecules we detected 2777 molecules. Multivariate analyses confirmed that changes in the metabolomic profile were associated with myometrial invasion (Figure 3C and 3D and Supplementary Figure S2). As above, robustness of models was evaluated by permutation test (p=0.069 for negative ionized molecules) and random-forest analyses (overall error 0.22 for positive and 0.37 for negative ionization) (Supplementary Figure S1B). Further, and using only those molecules which have potential identity (based on exact mass, retention time, isotopic distribution and/or MS/MS spectrum), we performed a correlational analyses comparing those metabolites levels in MIF and SEC (Figure 4). This demonstrated that most of these metabolites did not show a significant correlation, reinforcing the idea of independent metabolism depending on the tumor's depth.

Then, we performed a paired T-Student Test and found 135 statistically different molecules between SEC and MIF. Apart from stearamide and monoolein, we could identify 11 molecules (Supplementary DataSet1). Among them, 7 were increased (xanthine, lactamide, alpha-D-Fucose, 3-mercaptopyruvate, ribitol, phosphocholine (32:0) and eicosapentaenoic) and 5 decreased (inosine, deoxycytidine, hypoxanthine, CDP-ethanolamine and 5′-methylthioadenosine) in MIF respect SEC location. Because most of these metabolites belong to purine metabolism we wanted to further explore the implication of this pathway in tumor myometrial invasion. Thus, we focused in the study of two different enzymes which regulate levels of xanthine, hypoxanthine and inosine. Specifically, we analyzed, using immunohistochemistry, xanthine oxidase and purine nucleoside phosphorylase levels of these samples but no changes were observed (data not shown).

## DISCUSSION

During endometrial tumorigenesis, cancer cells have to remodel cellular metabolism to cover their demands of growth and proliferation. Although many of the metabolic changes are analogous to those seen in normal proliferative cells, studies over the past years have shown that many features of transformed tumors cell metabolism lie directly downstream of several tumor suppressors and oncogenes [15]. However, altered tumor metabolism is not the simply result of some combination of cell-autonomous genetic alterations. Tumor microenvironment parameters, such hypoxia, have to be considered as a component in the equation that influences changes in tumoral metabolism [16]. Knowledge of these metabolic signatures can enable identification of novel therapeutic targets and more important, can reveal the metabolic pathways and specific targets in which intervention could be more effective in patient therapeutic approaches. Based on that, the first aim of the present work was the study of metabolomic differences between EEC and NE. Heatmap analyses showed that carcinogenic process defined a specific metabolomics profile. In order to better characterize these changes, we applied multivariate statistics, specifically partial least square discriminant analyses (PLS-DA). Multivariate statistic such as principal component analyses (PCA), PLS-DA or hierarchical clustering analyses are commonly used for interpretation and better visualization of complex data [17,18]. PLS-DA analyses reinforced the idea of profile of endometrial cancer, obtaining an out of bag error of 0,2. Then, we applied univariate statistics trying to describe novel metabolites and pathways potentially involved in this pathogenic process. Specifically, we could identify four metabolites, two of them related to endocannabinoid metabolism.

The endocannabinoid system is implicated in a variety of physiological and pathological conditions (inflammation, immunomodulation, analgesia, cancer and others). Endocannabinoids are endogenous lipid-signaling molecules generated in the cell membrane from phospholipid precursors and which produce its effects through activation of CR1 and CR2 receptors [13]. In tumor cells, several studies have described alterations in endocannabinoid system; however it remains unclear if the endocannabinoid system has an anti- or protumoral role [19, 20]. In agreement with other published results in other cancer types such as breast, prostate or colorectal cancers [21–23], our results suggest an implication of endocannabinoid pathway in endometrial cancer progression, because we demonstrate that the endocannabinoid-like ligands, such as stearamide and monoolein are increased in EC samples. Previous data in humans reveal that stearamide levels are increased in serum from cyrrothic patients (either considering alcohol or hepatitis B virus caused ones) [24]. This was attributed to an increased activation of sterol-regulatory-element- binding protein 1, a key regulator of lipogenic genes. Since this protein has been involved in the proliferation inhibition and apoptosis in endometrial cancer [25], it may be hypothesized that increased stearamide could be a response towards EC by healthy cells. In this line, EC cells could take profit by increasing endocanabinoid receptors.

Previous studies in a small series of EC have shown higher CR2 levels [26]. In our series (60 normal tissue, 62 EEC), expression of endocannabinoid receptor 1 is significantly raised in endometrial carcinoma. Furthermore, CR2 shows a higher expression in III and IV stages, suggesting that endocannabinoid receptor 2 expression could be associated with poor prognosis. In this context, the potential disturbance of endocannabinoid metabolism in cell differentiation, growth and migration related to EC etiopathogenesis needs to be further studied.

Histological grade is a good predictor of aggressive behavior. Grade 3 EEC is an aggressive tumor, with increased frequency of lymph node metastasis. It represents 15% of EEC, but accounts for 27% of cancer deaths. In our study, we have identified different metabolomic signature depending on EEC grade arising an endocannabinoid (oleamide) as potential biomarkers of tumor aggressive behavior. Future studies are needed to disclose the importance of endocannabinoid metabolism in tumor aggressive behavior.

There are increasing evidences that tumors are composed of subpopulations of cells with distinct genomic alterations. With the advent of last generation sequencing techniques, the extent and prevalence of intra-tumor heterogeneity is becoming acknowledged. It is now clear that there is a competition between microclones of cancer cells, which have differential molecular features and tumor stromal interaction capability [6]. Intratumor heterogeneity is also seen when comparing tumor cells at the invasive front with cells located in tumor surface. Previously, our group compared samples from the surface area and the myoinvasive front of EEC in order to investigate whether the EMT (epithelial mesenchymal transition) program is activated in early stages of EC. We found increases in SLUG, ZEB1, and HMGA2 mRNA expression in the myoinvasive front of tumor samples, indicating the role of these transcriptional factors in endometrial tumor progression and invasion [6]. Also related to EMT, ETS transcription factors have been associated with the activation of matrix-degrading proteases. Specifically, up-regulation of ERM/ETV5, a member of the ETS transcription factors, has been recently associated with initial steps of myometrial invasion in EC, in correlation with increased matrix metalloproteinase (MMP) [27]. Furthermore, our group used a proteomic approach to characterize specific components of the invasive front by comparing the invasive area of a tumor with pure tumor and normal tissue from the same patients. Of interest resulted the identification of different enzymes involved in homeostasis of oxidative stress such as SOD1 or BLVRB2 [28]. Interestingly, oxidative stress target downstream molecules to trigger tumor progression, and are expected to have consequences in tumor metabolism [29, 30]. Consequently, changes in metabolism of tumoral cells, as well as its heterogeneity could be important in tumor prognosis, accounting responses to different oxygen and nutrient requirements.

The present results reveal the existence of intratumor heterogeneity, also at the metabolic level, when comparing tumor cells at the invasive front with the cells that are located in the surface of the tumor. Both multivariate and univariate statistics indicated that there is a specific metabolomic signature of invasive front when it is compared with the surface. So, using metabolomics we could define not only a specific signature for EEC in comparison with NE, but also discriminate EEC depending on the invasion (SEC, MIF), or grade. Interestingly, monoolein also arose as a potential biomarker of MIF zone. To further explore whether cannabinoid metabolism was implicated in myometrial invasion, we evaluated stearamide and monoolein levels comparing 3 groups (NE, SEC and MIF). Surprisingly, the two endocannabinoid-like metabolites presented different tendency in MIF, having higher levels of stearamide but lower levels of monoolein. Again, these results suggest an implication of endocannabinoid system in EC pathogenesis, although its effects are still unclear. Pathway analyses revealed different levels of metabolites belonging to purine metabolism when invasive front and surface zone were compared. The deregulation of purine metabolism in cancer has been widely described in human samples and cell lines [9, 31], but in this paper we described for the first time 3 metabolites belonging to purine metabolism (inosine, xanthine, hypoxanthine) which are increased at the invasive front in EEC.

In conclusion, metabolomics technique could contribute to further understand the pathogenesis of EC but also could shed light in the processes involved in myometrial invasion, proposing new targets for possible therapeutic intervention.

## MATERIALS AND METHODS

### Patient selection and sample collection

For metabolomics fresh-frozen tissue samples were obtained, corresponding to 15 normal endometrium (NE) (10 proliferative and 5 secretory) and 27 EEC (6 grade 1, 13 grade 2, 8 grade 3). For each of these EEC cases, two different samples were dissected, one from SEC and one from MIF. For immunohistochemical analysis, formalin-fixed, paraffin-embedded (FFPE) tissue samples were obtained including 60 samples of NE (38 proliferative, 22 secretory), and 62 cases of EEC (21 grade 1, 35 grade 2, and 6 grade 3). Fifty tumors were stage I, 8 tumors stage II, 2 tumors stage III and 2 tumors stage IV. FFPE sections were microscopically reviewed and representative areas of the tumors were selected in the corresponding blocks. A Tissue Arrayer device (Beecher Instruments, Sun Prairie, WI, USA) was used. Two selected cylinders (0.6mm in largest diameter) were identified in each one of the 62 tumors (124 samples), which, in 28 of them, were known to be obtained from tumor surface and deep myoinvasive front, respectively. All samples were obtained from the surgical pathology files of Hospital Universitari Arnau de Vilanova de Lleida, and the biobank of IRBLLEIDA. A specific informed consent was obtained from each patient in both groups, and the study was approved by the ethical committee of the Universitari Hospital Arnau de Vilanova (Lleida, Spain).

### Metabolite extraction

Tissue samples were homogenized in a buffer containing 180 mM KCl, 5mM 3-[N-morpholino]propanesulfonic acid, 2 mM ethylenediaminetetraacetic acid (EDTA), 1 mM diethylenetriaminepentaacetic acid and 1 mM butylated hydroxyl toluene, 10 mg/ml aprotinin, 1 mM phenylmethylsulfonyl fluoride, pH 7.3 with a Potter– Eljeveim device at 4°C. Protein concentration was measured using the Lowry assay (Bio-Rad Laboratories, München, Germany) with bovine serum albumin used as a standard. 100 μg of each sample was resuspended in a total of 30μl of homogenizing buffer and vortexed for one minute. In order to extract metabolites from tissue homogenates, 90 μL of ice cold methanol was added to each sample, incubated at −20°C for 1h and centrifuged at 12000g for 3 min at room temperature, as described elsewhere [32]. The supernatant were recovered, evaporated using a Speed Vac (Thermo Fisher Scientific, Barcelona, Spain) and resuspended in water containing 0.4% acetic acid and 2 ng/ml of deuterium labelled docosahexaenoic acid (d4-DHA) in methanol as internal standard (1:1, v/v).

### Metabolome analysis

For the metabolomic studies, an Agilent 1290 LC system coupled to an ESI-Q-TOF MS/MS 6520 instrument (Agilent Technologies, Barcelona, Spain) was used. In all cases, 2 μL of extracted sample was applied onto a reversed-phase column (Zorbax SB-Aq 1.8 μm 2.1 × 50 mm; Agilent Technologies, Barcelona, Spain) equipped with a precolumn (Zorba-SB-C8 Rapid Resolution Cartridge 2.1 × 30mm 3.5 μm; Agilent Technologies, Barcelona, Spain) with a column temperature of 60°C. The flow rate was 0.6 mL/min. Solvent A was composed of water containing 0.2% acetic acid and solvent B was composed of methanol 0.2% acetic acid. The gradient started in 2% B and increased to 98% B in 13 min and hold at 98% B during 6 min. Pot-time was established in 5 min.

Data were collected in positive and negative electrospray mode TOF operated in full-scan mode at 50–1600 m/z in an extended dynamic range (2 GHz), using N2 as the nebulizer gas (10 L/min, 350°C). The capillary voltage was 4000 V with a scan rate of 1.5 scan/s. The ESI source used a separate nebulizer for the continuous, low-level (10 L/min) introduction of reference mass compounds: 121.050873, 922.009798 (positive ion mode) and 119.036320, 966.000725 (negative ion mode), which were used for continuous, online mass calibration. The MassHunter Data Analysis Software (Agilent Technologies, Barcelona, Spain) was used to collect the results, and the MassHunter Qualitative Analysis Software (Agilent Technologies, Barcelona, Spain) was used to obtain the molecular features of the samples, representing different, comigrating ionic species of a given molecular entity using the Molecular Feature Extractor (MFE) algorithm (Agilent Technologies, Barcelona, Spain), as described [33, 34]. Finally, the MassHunter Mass Profiler Professional Software (Agilent Technologies, Barcelona, Spain) was used to perform a nontargeted metabolomic analysis of the extracted features. We selected samples with a minimum of 2 ions. Multiple charge states were not considered. Compounds from different samples were aligned using a RT window of 0.1% ± 0.25 min and a mass window of 20.0 ppm ± 2.0 mDa. Only common features found in at least 50% of the samples of the same condition were considered, correcting for individual bias. The masses with significant differences (T-Test, p< 0.05) in abundance were searched against METLIN Metabolite PCD/PCDL (Agilent Technologies, Barcelona, Spain). Identities of metabolites were attributed based on an ortogonal approach: both identical chromatographic behaviour (RT+- 0.5 min) and identical m/z (<10 ppm) with those molecules present in the PCDL database. After that, we confirmed the proposed identities proposed based on MS/MS spectrum obtained in independent runs. All abundances were adjusted to the internal standard, ionisable in both positive and negative mode (d4-DHA). Partial least discriminant analyses (PLS-DA), receiver operating characteristics analyses and random forest analyses were done using Metaboanalyst Software [35].

### Immunohistochemistry

To validate the obtained results, we analyzed tissue microarray blocks, which were sectioned at a thickness of 3 mm, dried for 1 h at 65°C before pre-treatment procedure of deparaffinization, rehydration and epitope retrieval in the Pre-Treatment Module, PT-LINK (DAKO) at 95°C for 20 min in 50x Tris/EDTA buffer, pH 9. Before staining the sections, endogenous peroxidase was blocked. Antibodies used were: cannabinoid receptor 1 (CR1) (1:100 dilution; LSBio), CR2 (1:100 dilution; Thermo Scientific), Human Purine Nucleoside Phosphorylase (1:1000 dilution; R&D Systems), Anti Xanthine Oxidase (1:100 dilution; Abcam). After incubation, the reaction was visualized with the EnVision Detection Kit (DAKO) using diaminobenzidine chromogen as a substrate. Sections were counterstained with hematoxylin. Appropriate positive and negative controls were also tested. Immunohistochemical results were evaluated by two pathologists, by following uniform preestablished criteria. Immunoexpression was graded semiquantitatively by considering the percentage and intensity of the staining. A histological score was obtained from each sample, which ranged from 0 (no immunoreaction) to 300 (maximum immunoreactivity). The score was obtained by applying the following formula: Histoscore= 1 x (% light staining) + 2 x (% moderate staining) + 3 x (% strong staining). The reliability of such score for interpretation of immunohistochemical staining in EC TMAs has been shown previously [36].

### Statistics

Statistical analyses for nontargeted metabolomics were done using the Mass Profiler Professional Software (Agilent Technologies, Barcelona, Spain). Otherwise, statistics calculations were performed using the SPSS software (SPSS, Chicago, IL) or the Stata 11 statistics package (StataCorp, College Station, TX). Normal distribution of the variables was checked by the Kolmogorov–Smirnoff test. Correlation between molecular features was evaluated by the Pearson correlation (PC) coefficient. Differences between different groups were analyzed by the Student's t test, ANOVA, Mann-Whitney or the Fisher test in the case of correlation matrixes. A p < 0.05 level was selected as the point of minimal statistical significance in every comparison.

## SUPPLEMENTARY FIGURES




