# Supplementary material for: Metabotyping human endometrioid endometrial adenocarcinoma reveals an implication of endocannabinoid metabolism
Source: Oncotarget. 2016 Jul 13;7(32):52364–74. doi: 10.18632/oncotarget.10564 (PMC5239558; doi:10.18632/oncotarget.10564)
Supplement: Supplementary file 1 [file oncotarget-07-52364-s001.pdf]

Metabotyping human endometrioid endometrial adenocarcinoma reveals an implication of endocannabinoid metabolism

SUPPLEMENTARY FIGURES

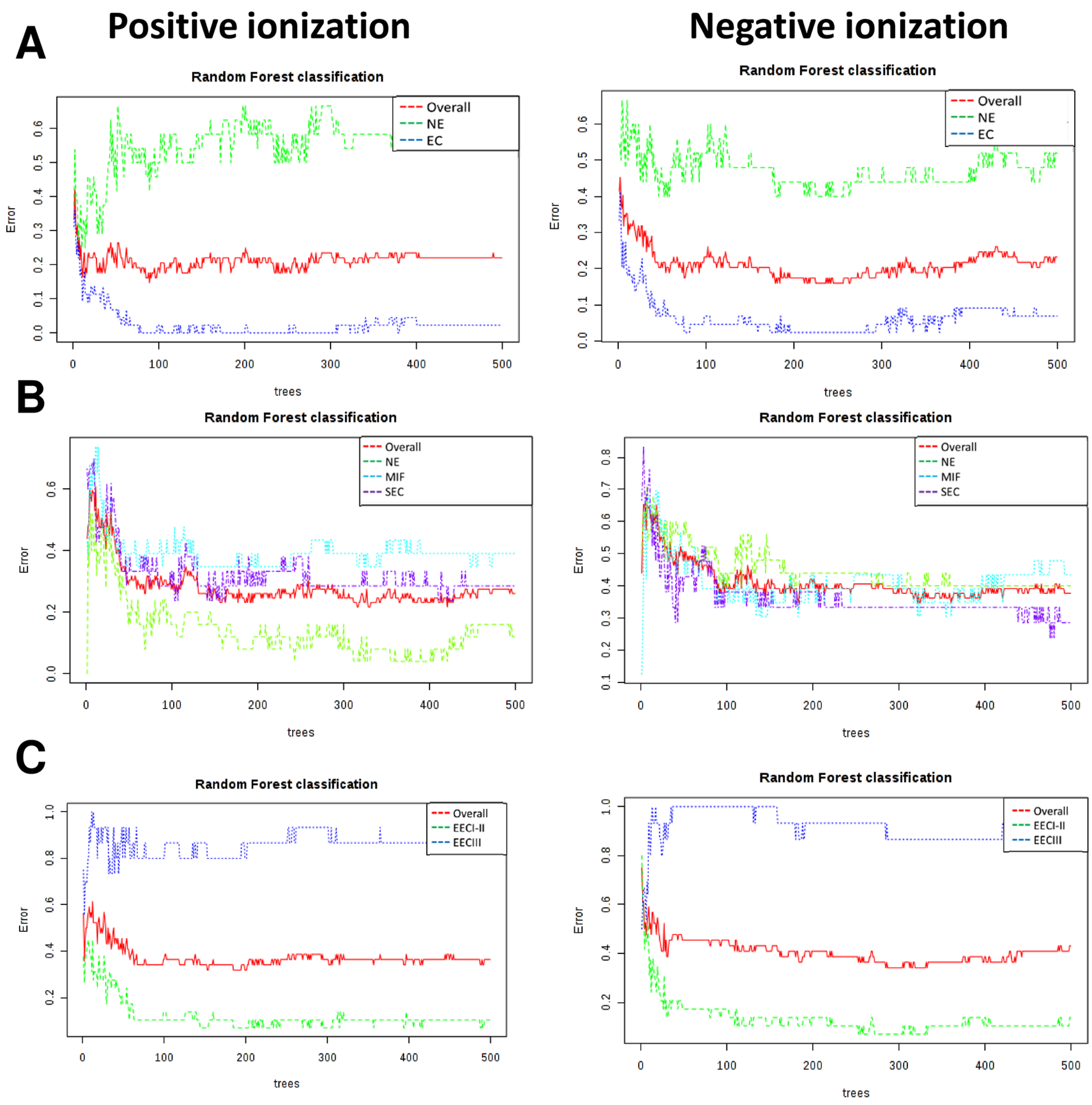

Supplementary Figure 1: Random forest classification for different studies included in the study.

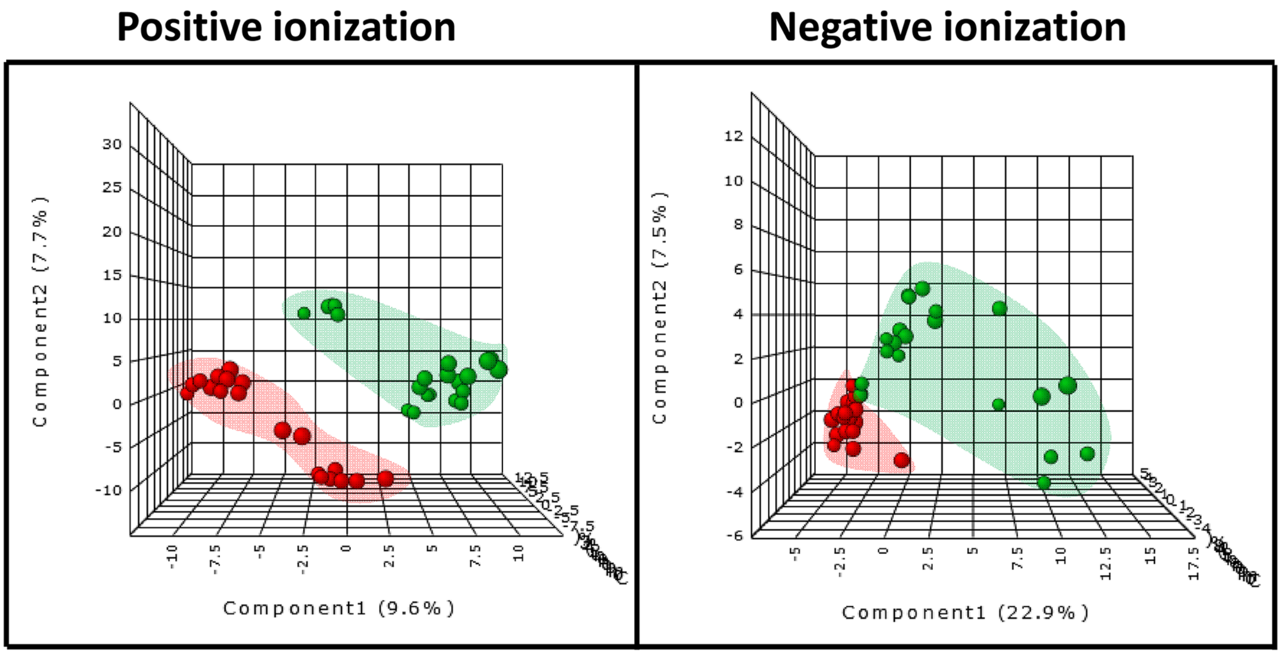

Supplementary Figure 2: PLS-DA graphs demonstrating the differentiation effect of location in metabolomic profiles. Red spots represent samples from myometrial invasive front and green spots surface endometrial carcinoma. PLS-DA model out of bag error is 0.195 for positive and 0.214 for negative ionization.

Supplementary Dataset 1: Statistically different molecules (Student T Test, $p<0.05$) between endometrioid endometrial carcinomas (EEC) and normal endometrium (CTL).

See Supplementary Dataset File 1
